# Sister Olive: A Hospital Romance

**Published:** 1886-12-18

**Authors:** W. J. Eccott


### Sister Olive: A Hospital Romance.

By W. J. Eccott.

Chapter IV.

"Better! much better!" said Hargen. "Doctor! You've been a brother to me. I don't think I shall die after all. But three days more in that Long Row must have finished me. And here I can breathe; the landlady's a treasure and I owe it all to you—well! There ......I want to thank you"...... It seemed as if he couldn't say more.

"Never mind the thanks till you get well," said Grant. "I can see you're a great deal better, but your weakness will last some time. Your system has been shaken much. Have you any idea how you came by the fever?"

"I was coming home from Egypt," said Hargen. "You must know I've been working as an engineer on some new railway works out there for a while, but what with drink and living utterly careless of what I did, my health broke down. So I threw up my appointment and came home. I came ashore at Marseilles and there I got worse; the hot weather and the stench upset me, and, by the time I'd finished the long railway journey through from Marseilles, I was down with fever."

"Whatever possessed you to drink in a hot climate like Egypt?" Grant asked.

"Mere devilment, I expect," said Hargen bitterly. "The fact is, for the last few years I haven't cared a bit what became of me. My constitution seemed made of iron. This is the first time it has really failed me. But I suppose the previous strains had weakened it. I've had some experiences, if any man has. It seems to me that when a fellow makes one slip, down he goes. Everyone gives him a helping kick. Ten years ago I was worth three thousand pounds. To-day I haven't a penny. That's running down pretty fast, isn't it? My people are what would be called wealthy. They live in Norfolk. My father made his pile by his profession as an engineer by hard work,—'early to bed, early to rise' business, you know. Then he retired and bought a country house and small estate. There was myself, the only son, and my sister. I was sent to an expensive school, where I fell in with Lumley. He hadn't much money, but he was very proficient as a sponge, and a grand tutor in the art of spending. Under his tuition I became a careless, idle fellow. When I left there it was my father's idea I should go into the army. I was sent to Blackheath to a tutor, to cram for the exams. The only cramming I did was to join the fastest set there and learn to play billiards and nap; to bet and drink and spend roaring nights up in town, which I ought to have spent in hard reading. Lumley was at Guy's then, studying medicine, and he introduced me to a set of medicals, whose object in joining a hospital it was difficult to guess. They never seemed to read. The Alhambra, the 'Cri.,' the 'Pav.,' as they called them, were their nightly resorts. More than the outside of an Anatomy Book I can't think they ever saw. They assisted me to see "life" and to spend money. Just about then I was fool enough to introduce Lumley to my people. At my father's house he was made welcome. He played the hypocrite better than I did. My father and mother found out pretty much how matters stood with me. My father, proud of his own industry, gave me hot and strong lectures, which I listened to with indifference and paid no heed. I was already imbued by my associates with a contempt for 'governors.'

"My mother was much younger than my father, and too much the fine lady to show real love. My sister was a mere school-girl then. If she was at home, when I paid a visit there, I snubbed her more or less. Other fellows' sisters interested me much more. But I often recall now dozens of affectionate things she did for me, the interest she would take in a football match, or a day's shooting, or anything else in which I took part. Lumley used to make a fuss of her. To my father and mother he was always very deferential and obliging, making his manners stand out in very agreeable contrast to mine, and not without hinting pretty frequently of my many delinquencies. I believe he made himself out—sneak, backbiter, and tempter that he was—to be a sort of guardian angel to me. Of course I didn't see his game. I gave him credit for being a decent fellow. Well, two years passed. I left one crammer for another. They took the fees, and, to do them justice, tried their best to get me to work; but it was of no use. I'd got into the way, and I went the pace along it. Now and then I received a strong letter of remonstrance from my father, or he came up and gave me a lecture. That only made me worse. Whenever she saw me, my sister would try to get to my heart by making me presents, sending me some of her pocket money if she thought I was hard up, and tried to keep things pleasant at home. I paid no heed to her: took all her worship as a right; spent the money in dissipation, as if it were not the sacred gift it was. I learned to be artificially pleasant to her, because I fancied it would pay. Sometimes I had letters from her—such letters, full of love and sympathy and gentle advice. Through all my ups and downs, if you believe me, Doctor, I've kept some fifty of those letters. I hadn't the heart to destroy them. I've been looking them over again these last few days, and I've been wondering how it was I couldn't then see what she was to me as I do now. But I'm tiring you, sir, with this history, surely. Wonderful, how a man can run on when he gets on to that interesting subject—himself."

"Tired?" said Grant. "Not a bit. A life-history like this is deeply interesting, I assure you. But here's Mrs. Hussey with your beef-tea."

Mrs. Hussey, all smiles, brought in a small tray, covered with a clean white cloth, whereon was a basin of hot beef-tea and some crisp, dry toast, which she set down before Hargen.

"Thanks," said Hargen. And then, turning to Grant as she went out—"Here's luxury! Fancy a clean cloth and beef-tea like this at Mrs. Jupp's, eh?"

"There, don't talk any more now," said Grant. "Get to work on that beef-tea. I declare I begin to feel hungry myself. I wonder if Mrs. Hussey can make a cup of coffee? I'll ring."

"Coffee, sir? Oh, yes, sir! I won't keep you waiting more than two or three minutes. The kettle is on!"

And very soon she brought in hot milk and coffee, which sent out a delightful fragrance, and some thin slices of bread and butter. Grant and Hargen—these strangely-assorted board companions—were as cheerful as could be. Nothing assists to break down the barriers of conventionality between men so much as to eat together.

In the course of a quarter of an hour, refreshed by his beef-tea, Hargen went on again, speaking of himself:—

"As you may guess, Doctor, I didn't pass my examination. The last chance had gone. Something suggested to me that I should try engineering. My father's influence, and a good premium, got me into one of the first firms in London. The novelty of the work, a new set of men, and perhaps a bit of natural turn for it, pulled me round for a time, and I was comparatively steady. But Lumley soon contrived to get me astray again. I went in more and more for betting. At last came one big day. I had the most certain tips, and I plunged rashly. At the end of the day I was the loser by five hundred pounds. My father came to hear of it—through Lumley, I suppose, for he was on more intimate terms with my people than ever. Though there was no lien upon him, or legal claim upon me, my father insisted on paying up every farthing for the sake of his honour. Then he cut me adrift, and wrote me a letter forbidding me to darken his doors again. I was mad, and wrote a stinging answer, telling him I didn't wish to. I knew it wanted but a few months until I came in for a small fortune of £3,000. Part of it—I hadn't reckoned how much—was already gone to the money-lenders. With the rest, when I got it, I went the pace freely. My irregularities at the works were winked at, as I worked pretty hard upon the whole, and I got through my time somehow. When that occurred my money was done too, and having assisted me to spend it, Lumley at last cut me, thinking his position in my home was secure. At my last half-sovereign, and not knowing exactly where to get the next, I joined an Irregular Cavalry Corps for service in South Africa. What a crew they were, to be sure! Half of them were of good family, or of good education; the other half old soldiers from all quarters of the globe—rolling-stones who had picked up information on all subjects except how to make a respectable stay-at-home citizen. Of course it was all right going up country. There were constant drills and riding-schools, and strict discipline was kept. But coming down was different. Drinking? There was any amount of it when the chance of getting it and the money came. And cards? A trooper would stake anything that the winner would be willing to take. And when we got our money and went aboard the troopship to come home, the gambling, night and day, was awful. Most of us lost every penny, and a few sharps got it all. I landed as poor as I went out. Who should meet me at the boat but my sister, now no longer a girl, but a lovely woman. She shewed the same affection, the same real delight to think I had come out scatheless. And at first I was glad to see her, proud to own her. But the feeling wore off. That soldier's life had roughened me, hardened me; and I found that I should not be received at home. I was glad to accept the money she gave me, when she found out, as she quickly did, how things were with me. Asking her questions about home, I gradually drew out of her that Lumley, who had been paying her marked attentions some time, had proposed to her; that my father and mother favoured his suit, and had made my sister's life wretched by persecuting her. She had refused Lumley, out of distrust of his real character, but he still pursued her with attentions. I was mad at this. That the man who had systematically, as I began to think, ruined me, should cast his avaricious eyes at my father's fortune, and should propose to himself to reach it by sacrificing my sister's happiness through a calculated marriage, was too much for me to stand. Badly as I had taken care of my sister, I was hotly sensitive on a matter like that. I brooded over it. As luck would have it, I met him one night at the top of the Haymarket. I was just enough under the influence of drink to be easily inflamed to a fighting mood. I picked a quarrel and knocked him down. Within a few minutes, I was in Vine Street Police Station under a charge of assault. I wrote to my sister, who sent the requisite bail. That was the last touch with me. The next day I went down to the Engineering Works and got a letter of recommendation, with which I procured a berth as fourth engineer on a P. and O. boat. Since then I've knocked about, sometimes on sea, sometimes ashore, in different parts of the world. That last blow to her wounded spirit, my getting taken up, was too much for her. She saw me before my ship sailed, made me promise to write to her, wherever I was, at certain periods, and told me she was going to take up hospital work as a means of escaping the odious attentions of Lumley, and the friction, ever-increasing, at home. She has means of her own sufficient to be independent of them. By one of those extraordinary turns of fate one can't account for here, I'm brought into the very hospital where my own sister superintends a ward."

Grant had been listening almost breathlessly to the latter portion of the narrative, and, at the last words, burst out:

"Do you mean to say that Sister Olive is your sister?"

"Yes! that's what they call her here! and, now, after all these years of neglect on my side, and of devoted self-sacrifice on hers, I at last know her, know what she has done for me; and now, for her sake, I'm going straight. Even now she's gone home to try and effect a reconciliation. She would not have left me, but that I told her in whose hands I was, and she felt confidence in you."

Grant felt this last observation keenly. Hargen went on with great earnestness.

"Yes! I'm going straight, now! I'm not going to be a drag and disgrace on her any longer—the blot on her life, the cloud over every joy, the object of every thought, every prayer, every sacrifice. As for that Lumley . . . ."

He had over-estimated his strength, and leant back exhausted. It passed over, however, and Grant said: "There; be quiet; you must not excite yourself in this weak state. Now leave off talking for to-night, and go to bed. Perhaps I'll come up to-morrow and have another chat. Meanwhile—good-bye."

When Grant reached home about nine, there was a letter awaiting him in Sister Olive's handwriting. He opened it eagerly. To his surprise it was dated :—"The Birkholts, Fothercliffe," and it bore the date of the day. It ran :—

"Dear Mr. Grant,

"Archie Tenison is here at home, ill of blood poisoning, contracted at a post-mortem. I'm here to nurse him. It seems serious. Come, if you can, at once.—Yours truly,

"Olive Carleston."

(*To be continued.*)

